# Donor-Derived Cell-Free DNA in Antibody-Mediated Rejection: An Analysis of the Surveillance HeartCare Outcomes Registry

**DOI:** 10.1016/j.jchf.2025.102716

**Published:** 2025-10-22

**Authors:** Paul J. Kim, Amit H. Alam, Jeffrey J. Teuteberg, Kiran K. Khush, Sean P. Pinney, Richard K. Cheng, Amin Yehya, Jeremy Kobulnik, Kevin M. Pinney, Kris A. Oreschak, Christopher R. Ensor, Steve Fan, Marcus A. Urey, Palak Shah, Shelley A. Hall

**Affiliations:** a Department of Medicine, University of California San Diego, La Jolla, California, USA; b Leon H. Charney Division of Cardiology, NYU Grossman School of Medicine, NYU Langone Health, New York, New York, USA; c Division of Cardiovascular Medicine, Stanford University, Stanford, California; d Mount Sinai Fuster Heart Hospital, Icahn School of Medicine at Mount Sinai, New York, New York, USA; e Division of Cardiology, University of Washington, Seattle, Washington, USA; f Advanced Heart Failure Center, Sentara Heart Hospital, Macon and Joan Brock Virginia Health Sciences at Old Dominion University, Norfolk, Virginia, USA; g Medical Affairs, CareDx, Brisbane, California, USA; h Biostatistics, CareDx, Brisbane, California, USA; i Cardiovascular Genomics Center, Inova Heart and Vascular Institute, Fairfax, Virginia; j Department of Cardiology, Baylor University Medical Center, Dallas, Texas, USA.

**Keywords:** biopsy, cardiac transplant, molecular biomarker

## Abstract

**BACKGROUND:**

Donor-derived cell-free DNA (dd-cfDNA) has emerged as a biomarker for antibody-mediated rejection (AMR), but its performance characteristics have not been evaluated in a large contemporary heart transplant population.

**OBJECTIVES:**

The study aimed to characterize the incidence and timing of biopsy-proven AMR and evaluate the performance characteristics of dd-cfDNA for AMR.

**METHODS:**

The authors included 2,240 subjects from the SHORE (Surveillance HeartCare Outcomes Registry) registry transplanted between 2017 and 2022 with verified biopsy, dd-cfDNA, echocardiographic, and donor-specific antibody (DSA) data. They evaluated the performance characteristics of dd-cfDNA for AMR and the incidence of AMR in different clinical contexts.

**RESULTS:**

AMR was present in 2.6% of biopsies with significant variability depending on the clinical context: AMR occurred in 1.1% of biopsies with normal graft function and no DSAs vs 20.4% of biopsies with known DSA and graft dysfunction. In patients with neither DSA nor graft dysfunction, the incidence of AMR was 0.7% for dd-cfDNA levels <0.20%, 1.2% for levels between 0.20% and 0.49%, and 6.7% for dd-cfDNA levels ≥0.50%. In patients with known DSA but no graft dysfunction, the incidence of AMR was 1.4% for dd-cfDNA levels <0.20%, 4.8% for levels between 0.20% and 0.49%, and 15.5% for dd-cfDNA levels ≥0.50%.

**CONCLUSIONS:**

The authors document significant context dependent variability of AMR incidence and the utility of dd-cfDNA in predicting biopsy yield. These data complement prior studies on the interpretation of peripheral gene expression profiling and dd-cfDNA for rejection monitoring and should further obviate the need for surveillance biopsies. (Surveillance HeartCare Outcomes Registry [SHORE]; NCT03695601).

## CENTRAL ILLUSTRATION

Donor-Derived Cell-Free DNA in the Diagnosis of AMR

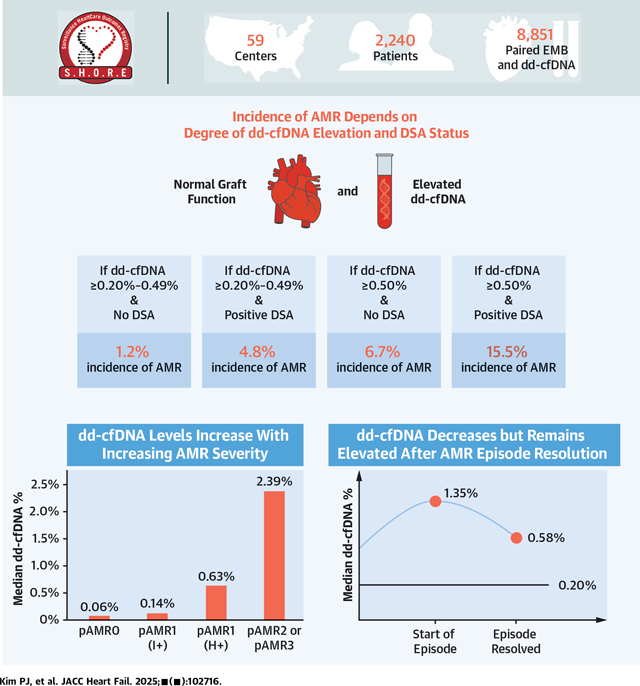

Antibody-mediated rejection (AMR) after heart transplantation is an important cause of allograft dysfunction and is associated with increased mortality and morbidity.^1^ Histologic and immunohistochemical staining on endomyocardial biopsy (EMB) is the current standard for diagnosing AMR, yet the diagnosis of AMR remains fraught with uncertainty.^2^ The reported incidence of AMR varies widely across transplant centers and has been attributed to differing surveillance practices, interobserver variability in pathologic interpretation, and whether abnormalities in histology, immunohistochemistry, or both are documented.^3,4^ Assessment for donor specific human leukocyte antibodies (DSA) often accompanies EMB; however, each program uses different mean fluorescence intensity (MFI) thresholds to determine their significance and the results vary both within and across laboratories.^5^ Last, unlike acute cellular rejection (ACR), there is no consensus on which findings should prompt treatment for AMR, which therapies are most efficacious, and which endpoints are indicative of a therapeutic response.^6^

We have previously shown that elevated levels of combined gene expression profiling and donor-derived cell-free DNA (dd-cfDNA) testing accurately identify risk of ACR.^7^ There is also some evidence that elevated levels of dd-cfDNA are associated with AMR and may even be higher during AMR than during ACR.^8–10^ To date, studies of dd-cfDNA for the diagnosis of AMR have been limited by a relatively small number of centers and of episodes of biopsy-proven AMR as well as by inconsistent incorporation of DSA findings and graft function.

Using the SHORE (Surveillance HeartCare Outcomes Registry) registry, our goals were to characterize the incidence, timing, and different pathologic grades of biopsy-proven AMR and to evaluate the performance characteristics of dd-cfDNA for the detection of biopsy-proven AMR.

## METHODS

### STUDY DESIGN AND PATIENT POPULATION.

The SHORE registry has been described previously.^7^ Briefly, the SHORE registry is a prospective, 67-center observational registry of adult heart transplant recipients monitored with gene expression profiling (AlloMap; CareDx) and dd-cfDNA (AlloSure; CareDx). The recommended gene expression profiling and dd-cfDNA testing schedule for the SHORE registry is provided in Supplemental Table 1. Clinical data, including EMBs, echocardiograms, and DSAs, were performed per standard of care and collected in the first 5 years post-transplantation using an electronic data capture system (Medrio; Medrio Inc). The registry sponsor (CareDx) is adjudicating the reports of key clinical variables, including EMBs, echocardiograms, coronary angiograms, and DSAs at all participating centers. As of October 2024, these key variables have been 100% verified, defined as review of all EMB, echocardiogram, coronary angiogram, and DSA reports in the electronic medical record from the date of transplantation up until 5 years post-transplantation, at 59 participating sites.

**FIGURE 1:**
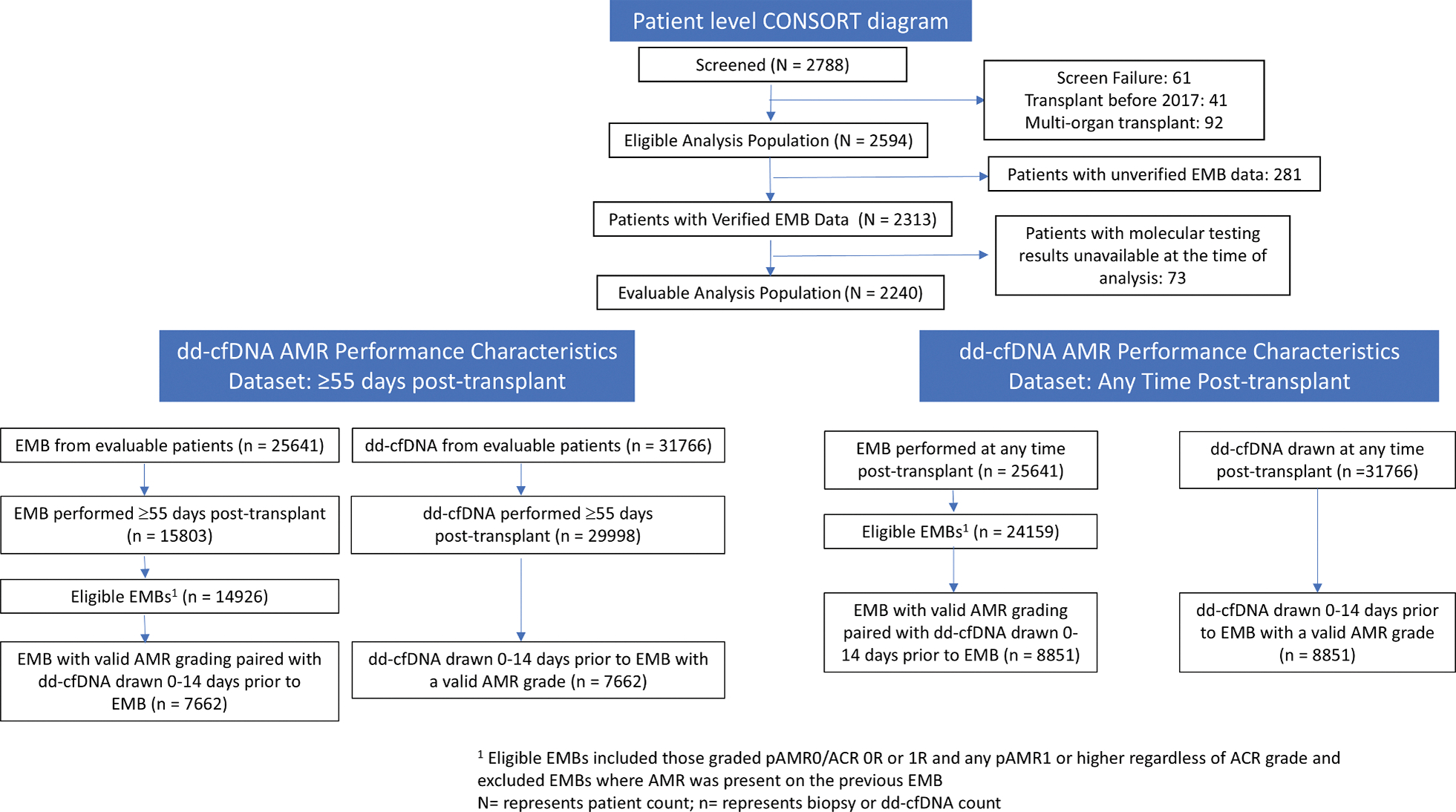
CONSORT Diagram ACR = acute cellular rejection; AMR = antibody-mediated rejection; dd-cfDNA = donor-derived cell-free DNA; EMB = endomyocardial biopsy.

This analysis included adult heart–only recipients transplanted between January 1, 2017, and December 31, 2022, from the 59 sites with complete clinical data available (Figure 1). Multiorgan recipients were excluded from this analysis. Patients who became pregnant were withdrawn from the SHORE registry. The decision to perform EMBs was determined by clinicians at each participating site. Consistent with prior cardiac transplant dd-cfDNA studies, continuous percent dd-cfDNA values were used for analysis, regardless of patient-level commercial reportable ranges that evolved during the study.^8,10^ The SHORE registry was approved by the Institutional Review Board at each of the 67 participating centers, and informed consent was obtained from each patient before enrollment.

### AMR INCIDENCE.

Biopsy-proven AMR was graded locally and defined as grade pAMR1 or higher according to the 2013 ISHLT (International Society for Heart and Lung Transplantation) grading system unless otherwise stated.^11^ dd-cfDNA and EMBs associated with ACR only were excluded from all analyses except the kinetics analysis. Mixed rejection (ie, ACR 2R or 3R and pAMR1 or higher) was considered AMR. Unless otherwise indicated, the calculation of AMR incidence excluded EMBs where the prior EMB demonstrated AMR. The incidence of biopsy-proven AMR was assessed overall and in 4 clinical scenarios: normal graft function/no known DSA, normal graft function/known DSA, graft dysfunction/no known DSA, and graft dysfunction/known DSA. A known DSA was defined as a positive DSA per each site on the most recent DSA assessment prior to the EMB. An additional analysis was performed where DSA positive was defined as requiring an MFI >5,000 due to the questionable clinical relevance of a weakly positive DSA.

### dd-cfDNA AND AMR DETECTION.

dd-cfDNA was considered paired with an EMB if drawn on the same day or up to 14 days prior to the EMB. If 2 EMBs occurred within 14 days of a dd-cfDNA draw, only the closest EMB was considered paired with the dd-cfDNA. dd-cfDNA levels were compared across AMR grades for all EMBs with a valid AMR result. Sensitivity, specificity and positive likelihood ratio (LR+) were calculated for dd-cfDNA at both 0.20% and 0.50% thresholds. In many instances, dd-cfDNA results did not prompt an EMB, which could affect the estimated dd-cfDNA performance characteristics for AMR. For example, patients with a low dd-cfDNA result and reassuring clinical picture may have their EMB deferred, potentially resulting in fewer negative biopsies being included in the performance characteristics analyses. As such, we performed a sensitivity analysis that only included EMBs and dd-cfDNA performed on the same day, where the result was not available to inform a clinician’s decision on whether to perform an EMB.

The biopsy-proven AMR incidence in each of the 4 aforementioned clinical scenarios was assessed for each of the following dd-cfDNA levels: <0.20%, 0.20% to 0.49%, and ≥0.50%. These thresholds were selected based on those used in previous publications on the performance of dd-cfDNA in heart transplant recipients.^8,9^ The aforementioned evaluations excluded EMBs in which the prior EMB demonstrated AMR. For example, if multiple consecutive EMBs were positive for AMR, only the first EMB and its paired dd-cfDNA would be included in the analysis. Given the possible confounders of primary graft dysfunction and ischemia reperfusion, these analyses were performed including and excluding EMBs/dd-cfDNA levels before 55 days post-transplantation. The 55-day timeframe was selected because that is when gene expression profiling becomes available, and precise performance characteristics of dd-cfDNA for AMR detection should be integrated with molecular detection of ACR. Taking into account differences in clinical practice for treating pAMR1 (I+), we conducted a sensitivity analysis in which pAMR1 (I+) was considered negative for biopsy-proven AMR.

Each patient had multiple dd-cfDNA tests paired with EMBs, which may potentially lead to an overestimate in the precision of performance characteristics. To account for this, a sensitivity analysis was conducted in which subject-level test performance metrics were calculated by averaging metrics estimated from each patient.^12^ To address the common clinical scenario of sequentially elevated dd-cfDNA, performance characteristics of dd-cfDNA for the detection of AMR were also calculated when the dd-cfDNA result immediately prior was elevated but associated with an EMB graded pAMR0.

Normal graft function was defined as a left ventricular ejection fraction of ≥50%. DSA positivity threshold, unless otherwise specified, was determined by each site and was defined as ≥1 DSA detected.

### KINETICS OF dd-cfDNA AFTER BIOPSY-PROVEN AMR AND ACR.

As an exploratory analysis, we evaluated the effect of presumed AMR treatment on dd-cfDNA levels by evaluating dd-cfDNA levels drawn ≥55 days post-transplantation and 0 to 14 days prior to either a new pAMR2/3 or a new pAMR1 with known DSA. This dd-cfDNA level was then compared with the dd-cfDNA level drawn on the same day or up to 60 days after the first negative EMB following the AMR event. For qualitative comparison, a similar analysis was conducted for ACR grade 2R or higher.

### STATISTICAL ANALYSES.

Descriptive statistics were used for patient demographics, clinical characteristics, and EMB results. Continuous variables were summarized as median (Q1-Q3) and categorical variables as frequencies, unless otherwise indicated. The 95% CIs for sensitivity and specificity were calculated using the Wilson interval method. LR+ was calculated as the ratio of sensitivity and 1 – specificity, and its corresponding CI was estimated based on the log transformation.

Between group comparisons for AMR incidence at different dd-cfDNA levels were performed using a chi-square contingency test. Mann-Whitney *U* with a Bonferroni correction for multiple testing was used to compare dd-cfDNA levels for each AMR grade. Change in dd-cfDNA levels after presumed treatment was assessed using a Wilcoxon signed rank test.

## RESULTS

Demographics and clinical characteristics of the 2,240 heart transplant patients included in this analysis are presented in Table 1. Patients were primarily White (65.8%) and male (73.6%), with a mean ± SD age at transplantation of 54 ± 12 years. Approximately one-third (31.2%) of patients received induction therapy and 20.2% had a calculated panel reactive antibody ≥10% at the time of transplantation. The median number of dd-cfDNA results per patient was 14 (Q1-Q3: 10–18), with patients followed for a median of 48.8 months (Q1-Q3: 40.8–57.5 months) post-transplantation. Patients with biopsy-proven AMR were more likely to be younger (*P* < 0.001), be female (*P* = 0.005), be Black (*P* = 0.016), and have a calculated panel reactive antibody ≥10% (*P* = 0.024) at the time of transplantation when compared with subjects who did not develop AMR.

### OVERALL INCIDENCE OF AMR.

**FIGURE 2:**
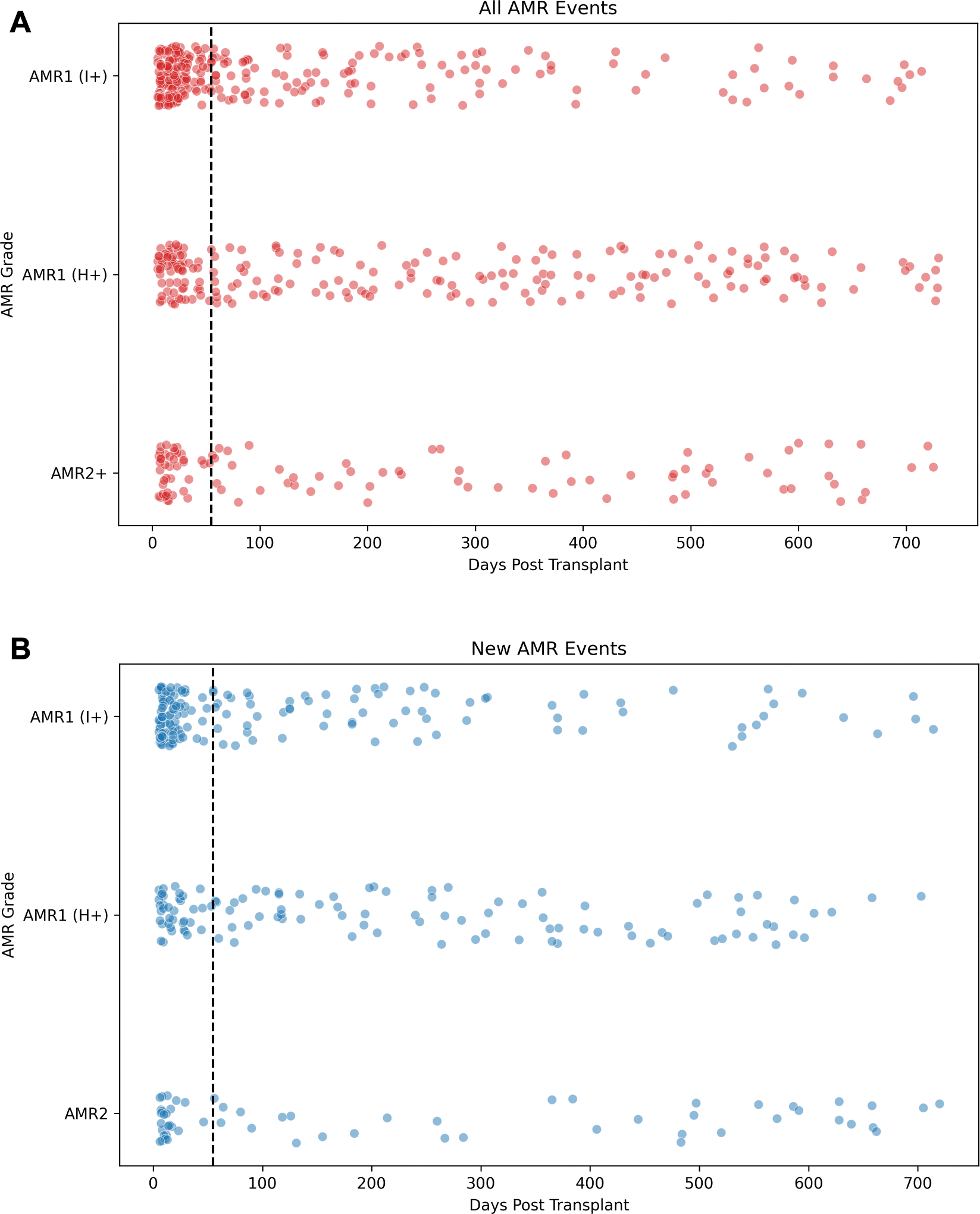
Biopsy-Proven AMR Grades by Time Post-Transplantation (A) All biopsy-proven AMRs, including those in which the biopsy immediately preceding it also demonstrated AMR. (B) Biopsy-proven AMRs excluding biopsies in which the biopsy immediately preceding it also demonstrated AMR. The dotted line represents 55 days post-transplantation. Abbreviation as in Figure 1.

Biopsy-proven AMR was present in 656 (2.6%) of 24,768 specimens, including EMBs in which AMR was present on the preceding EMB. pAMR1 (I+) was the most common result, occurring in 274 EMBs; pAMR1 (H+) was present on 262 EMBs, and 120 EMBs were graded pAMR2 or higher. Of the 656 EMBs with AMR, 266 (40.5%) occurred in the first 2 months post-transplantation, with more occurring in month 1 than in month 2 (203 vs 63) (Figure 2A).

Similarly, in 15,289 EMBs performed after 55 days post-transplantation, 409 (2.7%) had AMR; there were 131 cases of pAMR1 (I+), 198 pAMR1 (H+), and 80 pAMR2 or higher. When excluding EMBs performed in which AMR was present on the prior EMB, AMR was present on 250 (1.7%) of 14,926 EMBs (Figure 2B). When excluding EMB performed in which AMR was present on the prior EMB and pAMR1 I+ is considered negative, AMR was present on 170 (1.1%) of 15,048 EMBs.

### INCIDENCE OF AMR IN CLINICAL SCENARIOS.

The incidence of biopsy-proven AMR based on graft function and DSA status was 1.1% (95% CI: 0.9%-1.2%) for normal function/no known DSA, 2.1% (95% CI: 1.2%-3.7%) for abnormal graft function/no known DSA, normal graft function/known DSA was 4.3% (95% CI: 3.6%-5.2%), and finally abnormal graft function/known DSA was highest at 20.4% (95% CI: 15.3%-26.8%) as seen in Table 2. Similar results were observed when using an MFI threshold >5,000 for DSA positivity or treating pAMR1 (I+) as negative for AMR (Supplemental Tables 2 and 3, respectively). Specifically, the incidence of biopsy-proven AMR in patients with normal graft function and no known DSAs was 1.3% (95% CI: 1.1%-1.4%) when only considering an MFI threshold >5,000 as DSA positive and 0.6% (95% CI: 0.5%-0.7%) when pAMR1 (I+) was considered negative.

**FIGURE 3:**
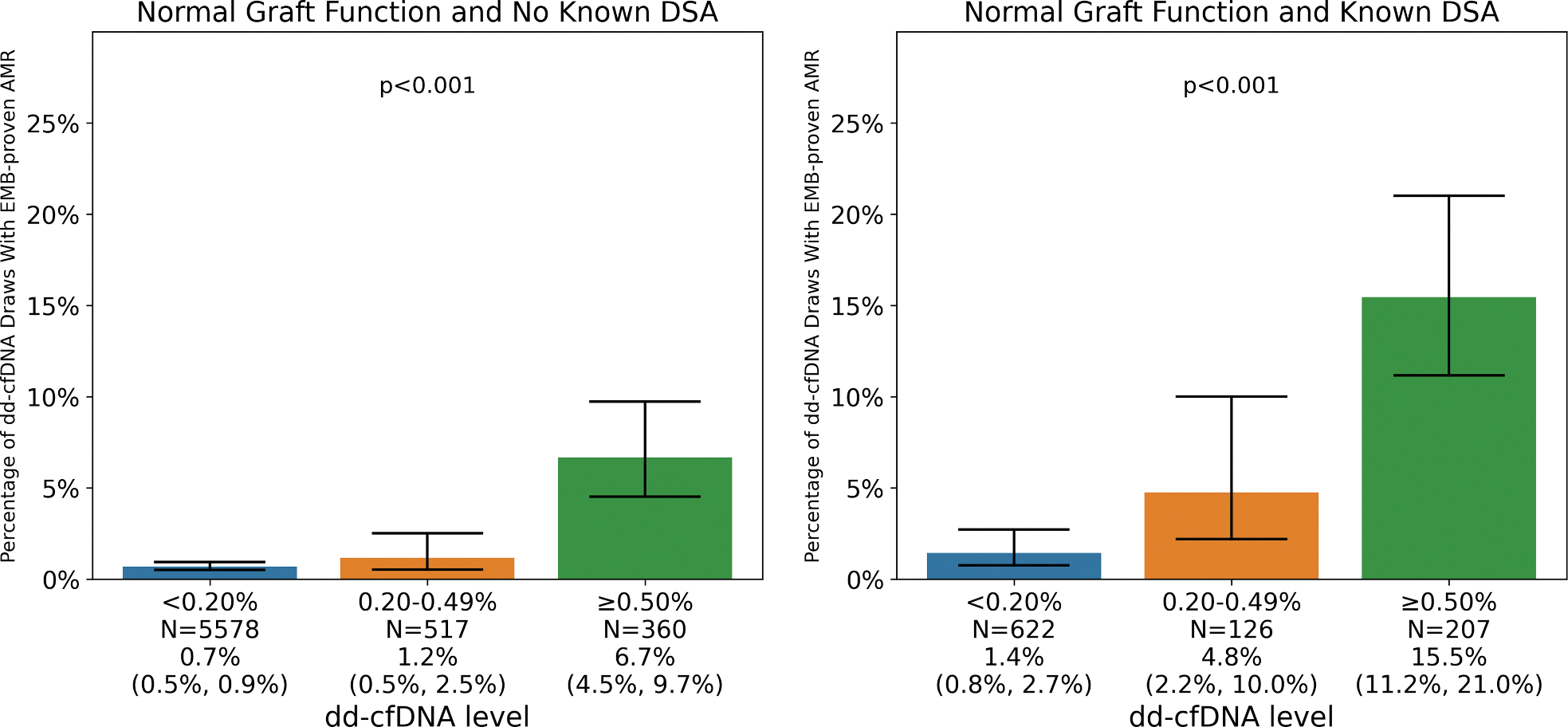
AMR Rates by dd-cfDNA Positivity in Select Clinical Scenarios Analysis included only EMBs and dd-cfDNA samples collected ≥55 days post-transplantation. Abbreviations as in Figure 1.

The incidence of biopsy-proven AMR by dd-cfDNA level in various clinical scenarios excluding paired samples before 55 days are provided in Figure 3 and Supplemental Figure 1. In general, the percentage of dd-cfDNA tests with an associated EMB increased with higher dd-cfDNA levels, with dd-cfDNA levels ≥0.50% having the highest EMB rates for all clinical situations, ranging from 33% in patients with abnormal graft function and known DSAs to 55% in patients with graft dysfunction but with no known DSAs. For patients with normal graft function and no known DSAs, the incidence of biopsy-proven AMR increased significantly with higher levels of dd-cfDNA (*P* < 0.001). The incidence of biopsy-proven AMR in patients with normal graft function and no known DSA was 0.7% (95% CI: 0.5%-0.9%) for dd-cfDNA levels <0.20%, 1.2% (95% CI: 0.5%-2.5%) for values between 0.20% and 0.49%, and 6.7% (95% CI: 4.5%-9.7%) for dd-cfDNA results ≥0.50%. In patients with normal graft function and a positive DSA, biopsy-proven AMR occurred in 1.4% of EMBs when dd-cfDNA levels were <0.20% (95% CI: 0.8%-2.7%), followed by 4.8% of EMBs for levels between 0.20% and 0.49% (95% CI: 2.2%-10.0%), and 15.5% of EMBs with levels ≥0.50% (95% CI: 11.2%-21.0%). The incidence of biopsy-proven AMR increased with higher levels of dd-cfDNA in the setting of abnormal graft function with both a negative DSA or a positive DSA (Supplemental Figure 1). Similar findings were observed for all scenarios when pAMR1 (I+) was considered to be negative for AMR (Supplemental Figure 2) and when paired samples before 55 days were included (Supplemental Figure 3).

### dd-cfDNA AMR PERFORMANCE CHARACTERISTICS.

Measurements of dd-cfDNA were available for 7,662 of 14,926 EMBs with a valid AMR grade performed ≥55 days post-transplantation (Table 3). The majority of dd-cfDNA paired with EMB were drawn in the first year post-transplantation (Supplemental Table 4); however, EMBs with paired dd-cfDNA were performed slightly later post-transplantation when compared with EMBs performed without paired dd-cfDNA (median 188 days [Q1-Q3: 114–359 days] vs 150 days [Q1-Q3: 83–339 days]; *P* < 0.001). In Black patients, 21.6% of EMBs were paired with a dd-cfDNA compared to approximately one-quarter (25.1%) of EMBs that were not paired with a dd-cfDNA (*P* < 0.001). Age at transplantation (56 years vs 55 years; *P* = 0.31) and sex (72.7% vs 72.2% male; *P* = 0.71) were similar between EMBs with and without paired dd-cfDNA. The incidence of AMR did not significantly differ between EMBs with and without a paired dd-cfDNA level (1.7% vs 1.7%; *P* = 0.95). Of 29,998 dd-cfDNA samples collected beyond 55 days post-transplantation, 22,336 samples did not have associated biopsies within 0 to 14 days. dd-cfDNA without paired EMBs were drawn later post-transplantation when compared with dd-cfDNA with a paired EMB (459 days [Q1-Q3: 247–813 days] vs 186 days [Q1-Q3: 113–357 days]; *P* < 0.001).

dd-cfDNA was significantly higher in the presence of biopsy-proven AMR when compared with EMBs negative for AMR (0.57% vs 0.06%; *P* < 0.001) (Table 3). Median dd-cfDNA levels paired with pAMR1 (I+), pAMR1 (H+), and pAMR2/3 were all significantly higher than levels paired with pAMR0. The dd-cfDNA values paired with an EMB with pAMR1 (H+) were significantly higher when compared with pAMR1 (I+) samples (0.63% vs 0.14%; *P* = 0.005). Last, EMBs graded pAMR2/3 had the highest dd-cfDNA levels of any group at 2.39% (95% CI: 0.74%-4.00%) and significantly higher than either pAMR1 (H+) or pAMR1 (I+) [*P* = 0.002 and <0.001, respectively). Similar results were found when including dd-cfDNA and EMB pairings at any time post-transplantation (Supplemental Table 5).

Performance characteristics of an elevated dd-cfDNA for detecting AMR on a subsequent EMB, excluding samples prior to 55 days, are presented in Table 4. At a threshold of 0.20%, the sensitivity of dd-cfDNA to detect any biopsy-proven AMR was 62.8% (95% CI: 54.2%-70.6%); specificity was 84.1% (95% CI: 83.2%-84.9%), and the LR+ was 3.94 (95% CI: 3.42–4.55). Using a threshold of 0.50%, the sensitivity of dd-cfDNA for any biopsy-proven AMR was 52.7% (95% CI: 44.1%-61.1%); specificity was 92.8% (95% CI: 92.2%-93.3%), and the LR+ was 7.29 (95% CI: 6.07–8.74). Similar results were observed for pAMR2/3 (Table 4). Similar performance characteristics were also observed when including samples prior to 55 days (Supplemental Table 6) or when including only dd-cfDNA and EMBs performed on the same day (Supplemental Table 7). Subject-level test performance characteristics were similar to results using sample-level data (Supplemental Table 8).

Finally, in subjects in whom the prior dd-cfDNA was ≥0.20% and was paired with an EMB negative for AMR, a sequential absolute increase of dd-cfDNA of at least 0.05% was associated with an increased risk of AMR, with an LR+ that ranged from ~2 to 9 (Table 5). An absolute increase of 0.20% was associated with an LR+ of 3.24 (95% CI: 1.74–6.04), while an absolute increase of 0.50% was associated with an LR+ of 6.64 (95% CI: 3.43–12.87).

### KINETICS OF dd-cfDNA FOLLOWING PRESUMED REJECTION TREATMENT.

**FIGURE 4:**
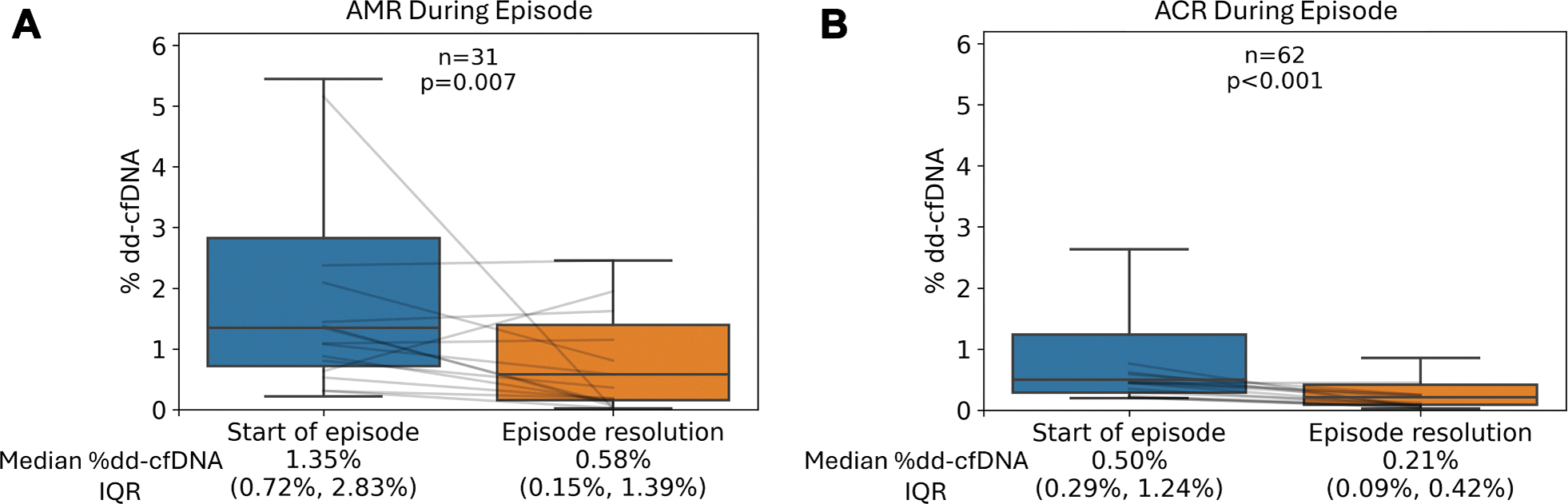
Decline in dd-cfDNA Following AMR or ACR Episode (A) This graph represents 31 AMR episodes in 28 patients. (B) This graph represents 62 ACR episodes in 57 patients. The figure includes only dd-cfDNA ≥0.20% at the start of episode and only EMBs and dd-cfDNA samples collected ≥55 days post-transplantation. Gray lines represent a random sampling of rejection episodes. Abbreviations as in Figure 1.

There was a significant decrease in dd-cfDNA in patients with pAMR2/3 or pAMR1 with known DSA, going from 0.87% at the time of the first EMB demonstrating AMR to 0.25% after the first negative EMB (*P* = 0.006). Similarly, levels of dd-cfDNA significantly decreased from 0.14% at the start of an ACR episode to 0.07% once the ACR episode resolved on EMB (*P* < 0.001) (Supplemental Figure 4). To account for the possibility of false positive EMBs and a consequent blunting of the presumed treatment effect, this analysis was repeated excluding initial EMBs without elevations in dd-cfDNA. When only rejection episodes with dd-cfDNA levels ≥0.20% at the start of the episode were included, dd-cfDNA for AMR fell from 1.35% to 0.58% (*P* = 0.007), whereas dd-cfDNA for ACR fell from 0.50% to 0.21% (*P* < 0.001) (Figure 4). The median time from the start of the rejection episode to resolution was 67 days (Q1-Q3: 32.5–133.5 days) for AMR and 19 days (Q1-Q3: 14–25 days) for ACR.

## DISCUSSION

In this large study of patients from the SHORE registry, including 2,240 patients and 8,851 EMBs paired with dd-cfDNA levels, we report the following key findings: 1) overall, the EMB yield for AMR is very low, especially when pAMR1 I+ is considered negative, and a large proportion of the EMBs demonstrating AMR occurred in the first 2 months post-transplantation; 2) elevations in dd-cfDNA were strongly associated with biopsy-proven AMR, with higher values correlating with higher pathologic grades of AMR; 3) rates of biopsy-proven AMR were very low for patients with normal graft function and no known DSA, even in the context of modest elevations of dd-cfDNA between 0.20% and 0.49%; 4) dd-cfDNA is more specific for AMR at a threshold of 0.50% (specificity of 92.8%) when compared with a dd-cfDNA threshold of 0.20% (specificity of 84.1%); and 5) there was a decline of dd-cfDNA after presumed treatment for biopsy-proven AMR, but it remained somewhat elevated, especially when dd-cfDNA was elevated at the onset (Central Illustration).

Although AMR is now widely recognized as a clinical entity, its frequency and the benefit of surveillance in detecting its subclinical form remain controversial. We have demonstrated that the overall EMB yield for AMR, especially when defining pAMR1 I+ as negative and excluding EMBs performed when the preceding EMB revealed AMR, was exceptionally low. Moreover, greater than one-third of AMR cases occurred in the first 55 days post-transplantation. This pattern raises the question of whether these early biopsy-proven AMR cases represent true rejection or if they may reflect alternate EMB findings due to microvascular and macrovascular injury or inflammation related to graft preservation and ischemia-reperfusion injury. Such injury patterns can mimic markers of AMR, potentially confounding the diagnosis in the immediate post-transplantation phase.^13^ Whether dd-cfDNA can be used early post-transplantation to identify which biopsy-defined AMR events represent true alloimmune-mediated graft injury requires further investigation.

Our study offers confirmation that dd-cfDNA is elevated in cases of AMR in a larger and more geographically diverse cohort than prior studies.^8–10^ In particular, patients with pAMR2 or pAMR3 demonstrated a median dd-cfDNA level of 2.39% (Q1-Q3: 0.74%-4.00%), a marked elevation compared with the levels of dd-cfDNA typically seen in cases of biopsy-proven ACR (range: 0.13%-0.34%).^8–10^

Biopsy-proven AMR rates varied based on graft function and DSA status, with a low incidence in patients with normal graft function and no DSAs but occurring more frequently in patients who were DSA positive. These findings support a nuanced and Bayesian approach to molecular test interpretation, suggesting that a single dd-cfDNA threshold to perform an EMB may not be universally applicable to all heart transplant recipients. Specifically, the use of a higher dd-cfDNA threshold of ≥0.50% may be practical when the pretest probability of biopsy-proven AMR is low, such as in patients with normal graft function and no known DSAs. In this same clinical situation, dd-cfDNA levels between 0.20% and 0.49% are unlikely to indicate biopsy-proven AMR. The use of other molecular tests in conjunction with dd-cfDNA, such as gene expression profiling, may help identify which of these modest elevations in dd-cfDNA are more likely to be associated with ACR, resulting in an even more targeted approach that further minimizes the number of EMBs. Finally, a lower dd-cfDNA threshold of 0.20% may be more appropriate in situations where there is a higher pretest probability of biopsy-proven AMR and when a positive EMB is also more likely to prompt treatment. For example, in the setting of a positive DSA, even a dd-cfDNA of between 0.20% and 0.49% has a 4-fold higher incidence of AMR when compared with the same dd-cfDNA range with no known DSA.

We note that in the setting of normal graft function and a known DSA, only 15.5% of patients with a dd-cfDNA level ≥0.50% had biopsy-proven AMR within 14 days. While we believe that this risk justifies an immediate EMB, it is possible that some of these patients develop AMR on a future EMB and may also merit closer follow-up. Previous work has shown that the risk of future clinical events, including rejection, is higher in this clinical context.^4,9^

A common clinical scenario that arises is when an elevation of dd-cfDNA leads to an EMB that is negative for AMR and is followed by another dd-cfDNA measurement, which is even higher than the initial result. These subsequent elevations of dd-cfDNA are associated with an increased risk of detecting AMR, albeit in many cases to a lesser degree than if not preceded by an elevated dd-cfDNA without rejection. It is possible the initial elevated dd-cfDNA associated with a negative EMB may have been an early manifestation of AMR, prior to identifiable histological changes or merely represented the limited sampling area of the EMB and the potentially patchy nature of AMR. This concept is supported by others who have documented a rise in dd-cfDNA as up to 3 months prior to abnormalities being detected on EMB.^9^

Intriguingly, dd-cfDNA levels were typically not elevated in cases of pAMR1 I+, with a median value of 0.14% (Q1-Q3: 0.04%-0.52%), indicating limited graft injury in many cases. This observation raises 2 important questions. First, in the absence of known DSAs or graft dysfunction, should low-grade AMR be treated, especially when dd-cfDNA elevations are minimal? Second, could dd-cfDNA serve as a tool to stratify the intensity or timing of treatment for AMR? For instance, a substantial rise in dd-cfDNA might indicate the need for more aggressive treatment, as it could signal a greater degree of underlying graft injury than what is apparent from EMB results alone. Ultimately, dd-cfDNA may provide a more accurate reflection of allograft injury, allowing clinicians to better contextualize EMB findings and more precisely tailor treatment decisions.

We analyzed the trajectory of dd-cfDNA following the presumed treatment of AMR and ACR. We included ACR in this portion of the analysis for comparative purposes and because it has never been previously quantified. In 31 patients with AMR and a dd-cfDNA ≥0.20% at the start of the AMR episode, there was a decrease in median dd-cfDNA from 1.35% to 0.58%, although dd-cfDNA levels often remained markedly elevated even after AMR resolution. dd-cfDNA levels also declined following presumed treatment for ACR; however, these values more frequently normalized upon resolution of ACR. There are several potential explanations for this: 1) some of the pAMR2/3 or pAMR1 with DSA episodes may not have been treated and the documented EMB resolution merely a reflection of inadequate tissue sampling; 2) the molecular response to treatment in AMR could be delayed, with persistent allograft injury lingering even after intervention; and 3) current treatment strategies for AMR may be insufficient to fully resolve the underlying graft injury. Given the reliability of dd-cfDNA as a marker of injury, it is likely that it will be used as an endpoint in future studies evaluating AMR treatment in cardiac transplant patients, as has already occurred in renal transplantation.^14^

### STUDY LIMITATIONS.

Although the registry collected comprehensive EMB results, the grading of rejection was performed locally without central adjudication. This introduces the potential for variability between centers in the interpretation of EMB results, leading to possible misclassification of AMR or its severity.^4^ Additionally, as the SHORE registry was a non-interventional study, each center independently determined the timing of dd-cfDNA testing, the threshold for EMB, whether or not to perform AMR staining, and the treatment decisions in response to abnormal results. Third, there was limited information collected on how sites adjudicated DSA positivity with varying MFI thresholds and no information regarding complement fixation and dilution. Notably, the findings for the locally determined DSA positive analysis were similar to those in which DSA positivity was defined as an MFI >5,000, suggesting that use of local thresholds did not significantly impact the results. Fourth, our analysis on the kinetics of dd-cfDNA following treatment of biopsy-proven AMR was limited by small sample size, heterogeneous response patterns, and assumed treatment, which was not collected as part of the SHORE registry. While it is possible that some rejection episodes were not treated, this was mitigated by including only pAMR2 or higher or any AMR associated with known DSA in the analysis.

Finally, an important limitation of the study is that not all EMBs were accompanied by dd-cfDNA and not all dd-cfDNA were accompanied by EMBs. This may have implications as to the generalizability of the results as well as the accuracy of the performance characteristics. That said, EMBs with and without associated dd-cfDNA shared similar features with only small differences in time post-transplantation and race of the recipient. With respect to accuracy of the performance characteristics, we are reassured by the very similar performance characteristics assessed in dd-cfDNA and EMBs performed on the same day. Moreover, it is true that the deferral of some EMBs after dd-cfDNA likely inflated the presented positivity rates; the lower the associated EMB rate was, the more inflated the result was, as lower-risk patients would be less likely to receive an EMB. However, given that the EMB rate was lowest for dd-cfDNA levels <0.20% and highest for dd-cfDNA levels ≥0.50%, the presented increase in AMR rates shown in Figure 3 are likely underestimated (rather than overestimated) by the analysis. Importantly, the presented data remain accurate as to what a clinician may expect to see in a real-world setting, as occurred in the SHORE registry.

## CONCLUSIONS

This is the largest contemporary real-world study that validates dd-cfDNA as a reliable biomarker of biopsy-proven AMR and specifies dd-cfDNA performance characteristics at different thresholds and in multiple common clinical scenarios. These findings complement the inaugural SHORE publication that focused on the use of molecular testing for ACR detection and will help centers build comprehensive strategies for interpreting molecular test results and further reduce the need for surveillance EMBs.^7^ Specifically, EMBs performed in response to dd-cfDNA levels between 0.20% and 0.49% without evidence of DSA or graft dysfunction are very unlikely to reveal AMR; in the absence of clinical or molecular evidence of ACR, these EMBs can mostly be avoided. Furthermore, given dd-cfDNA’s documented fidelity with EMB evidence of injury, combined with its resistance to sampling errors and subjective interpretation, there is a compelling case to incorporate dd-cfDNA into the definition of AMR and treatment decisions.

## Supplementary Material

supp

**APPENDIX** For supplemental tables and figures, please see the online version of this paper.

## Figures and Tables

**TABLE 1:** Demographics and Clinical Characteristics

	Total Cohort (N = 2,240)	Patients Without AMR (n = 1,969)	Patients With AMR (n = 271)	*P* Value

Age at transplantation, y	54 ± 12	55 ± 12	50 ± 14	<0.001
Sex				0.005
Female	591 (26.4)	500 (25.4)	91 (33.6)	
Male	1,649 (73.6)	1,469 (74.6)	180 (66.4)	
Race				0.016
Black	484 (21.6)	408 (20.7)	76 (28.0)	
White	1,474 (65.8)	1,315 (66.8)	159 (58.7)	
Other/unknown	282 (12.6)	246 (12.5)	36 (13.3)	
Reason for transplantation				0.079
NICM	1,420 (63.4)	1,246 (63.3)	174 (64.2)	
ICM	640 (28.6)	573 (29.1)	67 (24.7)	
Other/unknown	180 (8.0)	150 (7.6)	30 (11.1)	
Pretransplantation MCS				0.052
None	953 (42.5)	840 (42.7)	113 (41.7)	
LVAD	680 (30.4)	592 (30.1)	88 (32.5)	
tMCS	568 (25.4)	508 (25.8)	60 (22.1)	
Other/unknown	39 (1.7)	29 (1.4)	10 (3.7)	
Induction therapy (yes)	699 (31.2)	613 (31.1)	86 (31.7)	0.834
Sensitized at transplantation (PRA ≥10%)	453 (20.2)	384 (19.5)	69 (25.5)	0.024
dd-cfDNA tests per patient post-transplantation	14 (10–18)	14 (10–18)	16 (11–20)	<0.001
Length of post-transplantation follow-up, mo	48.8 (40.8–57.5)	49.1 (40.8–57.4)	47.2 (40.0–59.1)	0.701
Time to first dd-cfDNA level, mo	1.9 (1.0–3.8)	1.9 (1.0–3.8)	1.8 (1.0–4.0)	0.937

Values are mean ± SD, n (%), or median (Q1-Q3), unless otherwise indicated.

AMR = antibody-mediated rejection; dd-cfDNA = donor-derived cell-free DNA; ICM = ischemic cardiomyopathy; LVAD = left ventricular assist device; MCS = mechanical circulatory support; NICM = nonischemic cardiomyopathy; PRA = panel reactive antibody; tMCS = temporary mechanical circulatory support.

**TABLE 2:** Incidence of AMR in Select Clinical Scenarios

Graft Function	DSA	EMBs	AMR Incidence (95% CI),%

Normal	Negative	20,940	1.1 (0.9–1.2)
Abnormal	Negative	563	2.1 (1.2–3.7)
Normal	Positive	2,470	4.3 (3.6–5.2)
Abnormal	Positive	186	20.4 (15.3–26.8)

Normal graft function is left ventricular ejection fraction ≥50%; DSA positive is positive per site.

DSA = donor-specific antibody; EMB = endomyocardial biopsy; other abbreviation as in Table 1.

**TABLE 3:** dd-cfDNA Levels by AMR Grade in Samples Collected ≥55 Days Post-Transplantation

	EMBs Paired With dd-cfDNA	Median dd-cfDNA, %	Q1-Q3, %	*P* Value	Most Recent DSA Positive^a^	MFI in DSA Positive^a,b^	Most Recent DSA With MFI >5,000^b^

pAMRO	7,533	0.06	0.02–0.13	Ref.	955 (12.7)	2,597 (1,453–5,100)	211 (2.9)
Any AMR	129	0.57	0.09–1.66	<0.001	60 (46.5)	5,968 (3,000–14,000)	31 (25.6)
pAMR1	104	0.42	0.07–1.14	<0.001	42 (40.4)	5,968 (3,039–13,441)	23 (23.0)
pAMR1 (I+)	37	0.14	0.04–0.52	0.002	16 (43.2)	10,844 (5,844–15,209)	10 (30.3)
pAMR1 (H+)	67	0.63	0.11–1.46	<0.001	26 (38.8)	5,028 (2,938–11,570)	13 (19.4)
pAMR2 or pAMR3	25	2.39	0.74–4.00	<0.001	18 (72.0)	9,506 (3,238–14,000)	8 (38.1)

Values are n (%) or median (Q1-Q3), unless otherwise indicated.

a DSA positive per site.

b Excludes DSA positive samples with no reported MFI.

MFI = mean fluorescence intensity; Ref. = Reference; other abbreviations as in Tables 1 and 2.

**TABLE 4:** dd-cfDNA Performance Characteristics for AMR at 0.20% and 0.50% Thresholds After Day 55

AMR Classification	Sensitivity (95% CI), %	Specificity (95% CI), %	LR+ (95% CI)

AMR performance characteristics; 0.20% threshold			
Any AMR	62.8 (54.2–70.6)	84.1 (83.2–84.9)	3.94 (3.42–4.55)
pAMR1 (H+), pAMR2, pAMR3	70.7 (60.7–79.0)	83.9 (83.1–84.8)	4.40 (3.82–5.07)
pAMR2 or pAMR3	84.0 (65.3–93.6)	83.5 (82.7–84.3)	5.10 (4.26–6.09)
AMR performance characteristics; 0.50% threshold			
Any AMR	52.7 (44.1–61.1)	92.8 (92.2–93.3)	7.29 (6.07–8.74)
pAMR1 (H+), pAMR2, pAMR3	63.0 (52.8–72.2)	92.7 (92.1–93.2)	8.60 (7.21–10.25)
pAMR2 or pAMR3	80.0 (60.9–91.1)	92.2 (91.6–92.8)	10.30 (8.35–12.72)

Analysis included only endomyocardial biopsies and dd-cfDNA samples collected ≥55 days post-transplantation.

LR+ = positive likelihood ratio; other abbreviations as in Table 1.

**TABLE 5:** AMR Performance Characteristics for an Increase in dd-cfDNA When the Prior dd-cfDNA Was ≥0.20% and Was Not Associated With AMR

Absolute Increase in dd-cfDNA From Previous Value	Sensitivity (95% CI), %	Specificity (95% CI), %	LR+ (95% CI)

0.00	47.4 (27.3–68.3)	77.5 (74.3–80.4)	2.11 (1.29–3.45)
0.05	47.4 (27.3–68.3)	82.1 (79.1–84.7)	2.65 (1.61–4.36)
0.10	42.1 (23.1–63.7)	85.0 (82.2–87.4)	2.81 (1.61–4.90)
0.20	36.8 (19.1–59.0)	88.6 (86.1–90.7)	3.24 (1.74–6.04)
0.30	36.8 (19.1–59.0)	90.8 (88.5–92.7)	4.02 (2.14–7.57)
0.40	36.8 (19.1–59.0)	93.5 (91.4–95.1)	5.65 (2.95–10.83)
0.50	36.8 (19.1–59.0)	94.5 (92.5–95.9)	6.64 (3.43–12.87)
0.60	31.6 (15.4–54.0)	95.4 (93.6–96.7)	6.90 (3.29–14.48)
0.70	31.6 (15.4–54.0)	96.4 (94.8–97.5)	8.76 (4.09–18.76)
0.80	26.3 (11.8–48.8)	97.1 (95.6–98.1)	9.04 (3.81–21.40)
0.90	21.1 (8.5–43.3)	97.5 (96.1–98.4)	8.43 (3.16–22.54)
1.00	21.1 (8.5–43.3)	97.8 (96.4–98.6)	9.49 (3.50–25.70)

Abbreviations as in Tables 1 and 4.

## PERSPECTIVES

### COMPETENCY IN MEDICAL KNOWLEDGE:

The incidence of AMR is overall low but dependent on whether there are DSAs or graft dysfunction. dd-cfDNA levels increase with increasing grades of AMR.

### COMPETENCY IN PATIENT CARE AND PROCEDURAL SKILLS:

dd-cfDNA can be used to identify patients at high risk for AMR and for a decision to proceed with an EMB. Different %dd-cfDNA thresholds should be used, depending on the pretest probability of AMR.

### TRANSLATIONAL OUTLOOK:

As a reliable biomarker of AMR, incorporating dd-cfDNA into the definition of AMR may help standardize the diagnosis and facilitate studies evaluating AMR treatment.

## ABBREVIATIONS AND ACRONYMS

ACR: acute cellular rejection
AMR: antibody-mediated rejection
dd-cfDNA: donor-derived cell-free DNA
DSA: donor-specific antibody
EMB: endomyocardial biopsy
LR+: positive likelihood ratio
MFI: mean fluorescence Intensity
