# Supplementary material for: Donor-Derived Cell-Free DNA in Antibody-Mediated Rejection: An Analysis of the Surveillance HeartCare Outcomes Registry
Source: JACC Heart Fail. Author manuscript; Available in PMC 2025 Dec 23. (PMC12719789; doi:10.1016/j.jchf.2025.102716)
Supplement: supp [file NIHMS2122883-supplement-supp.docx]

## **Supplemental Table 1: Recommended SHORE donor-derived cell-free DNA and gene expression profiling testing schedule**

|  | **Time post-transplant** | | |
| --- | --- | --- | --- |
|  | **Year 1** | **Year 2-3** | **Year 4-5** |
| **dd-cfDNA timepoints (months post-transplant)** | 1, 2, 3, 4, 5, 6, 7, 8, 9, 10, 11, 12 | 15, 18, 21, 24, 27, 30, 33, 36 | 42, 48, 54, 60 |
| **GEP timepoints**  **(months post-transplant)** | 2, 3, 4, 5, 6, 7, 8, 9, 10, 11, 12 | 15, 18, 21, 24, 27, 30, 33, 36 | 42, 48, 54, 60 |

**Abbreviations:** dd-cfDNA, donor-derived cell-free DNA; GEP, gene expression profiling

## **Supplemental Table 2:** Incidence of antibody mediated rejection using an MFI threshold of 5000

| **Graft function** | **DSA** | **N= EMBs** | **AMR incidence** |
| --- | --- | --- | --- |
| Normal | Negative | 22855 | 1.3% (1.1%-1.4%) |
| Abnormal | Negative | 674 | 4.5% (3.1%-6.3%) |
| Normal | Positive | 555 | 8.1% (6.1%-10.7%) |
| Abnormal | Positive | 75 | 26.7% (18.0%-37.6%) |

**Normal graft function**: LVEF ≥50%; **DSA positive**: MFI >5000

**Abbreviations:** AMR, antibody mediated rejection; DSA, donor specific antibody; EMB, endomyocardial biopsy; MFI, mean fluorescence intensity

# Supplemental Table 3: Incidence of antibody mediated rejection [pAMR1 (H+), pAMR2, pAMR3] in select clinical scenarios

| **Graft function** | **DSA** | **N= EMBs** | **AMR incidence** |
| --- | --- | --- | --- |
| Normal | Negative | 21105 | 0.6% (0.5%-0.7%) |
| Abnormal | Negative | 569 | 1.4% (0.7%-2.7%) |
| Normal | Positive | 2557 | 3.0% (2.4%-3.7%) |
| Abnormal | Positive | 192 | 11.5% (7.7%-16.7%) |

**Normal graft function**: LVEF ≥50%; **DSA positive**: Positive per site

**Abbreviations:** AMR, antibody mediated rejection; DSA, donor specific antibody; EMB, endomyocardial biopsy

# Supplemental Table 4: Timing of dd-cfDNA draws paired with EMB

| **Time post-transplant** | **N= patients with a dd-cfDNA paired EMB^1^** | **n= dd-cfDNA paired EMBs** | **% of all dd-cfDNA paired EMBs^2^** |
| --- | --- | --- | --- |
| **55-90 days** | 988 | 1419 | 18.5% |
| **91-180 days** | 1252 | 2237 | 29.2% |
| **181-365 days** | 1191 | 2296 | 30.0% |
| **1-1.5 years** | 609 | 721 | 9.4% |
| **1.6-2 years** | 339 | 421 | 5.5% |
| **>2 years** | 383 | 568 | 7.4% |

^1^Patients could have a dd-cfDNA paired EMB at more than one time point

^2^Represents the percentage of all dd-cfDNA paired EMBs (n=7662) that were drawn in the respective time window

**Abbreviations:** dd-cfDNA, donor-derived cell-free DNA; EMB, endomyocardial biopsy

# Supplemental Table 5: dd-cfDNA levels by antibody mediated rejection grade in samples collected any time post-transplant

|  | **n=** | **Median dd-cfDNA** | **IQR** | **p-value** |
| --- | --- | --- | --- | --- |
| **pAMR0** | 8715 | 0.07% | 0.03%-0.15% | Reference |
| **Any AMR** | 136 | 0.49% | 0.09%-1.54% | <0.001 |
| **pAMR1** | 111 | 0.31% | 0.07%-1.03% | <0.001 |
| **pAMR1 (I+)** | 43 | 0.13% | 0.04%-0.46% | 0.005 |
| **pAMR1 (H+)** | 68 | 0.60% | 0.11%-1.57% | <0.001 |
| **pAMR2 or pAMR3** | 25 | 2.09% | 0.74%-4.00% | <0.001 |

**Abbreviations:** AMR, antibody mediated rejection; dd-cfDNA, donor-derived cell-free DNA; IQR, interquartile range

# Supplemental Table 6: dd-cfDNA performance characteristics for antibody mediated rejection at 0.20% and 0.50% thresholds including samples collected <55 days post-transplant

| **AMR performance characteristics; 0.20% threshold** | | | |
| --- | --- | --- | --- |
| **AMR classification** | **Sensitivity** | **Specificity** | **LR+** |
| **Any AMR** | 61.8% (53.4%, 69.5%) | 82.0% (81.1%, 82.8%) | 3.42 (2.98, 3.94) |
| **pAMR1 (H+), pAMR2, pAMR3** | 71.0% (61.1%, 79.2%) | 81.8% (81.0%, 82.6%) | 3.91 (3.41, 4.48) |
| **pAMR2 or pAMR3** | 84.0% (65.3%, 93.6%) | 81.5% (80.7%, 82.3%) | 4.53 (3.80, 5.41) |
|  | | | |
| **AMR performance characteristics; 0.50% threshold** | | | |
| **AMR classification** | **Sensitivity** | **Specificity** | **LR+** |
| **Any AMR** | 50.0% (41.7%, 58.3%) | 93.2% (92.6%, 93.7%) | 7.31 (6.08, 8.80) |
| **pAMR1 (H+), pAMR2, pAMR3** | 62.4% (52.2%, 71.5%) | 93.1% (92.5%, 93.6%) | 9.01 (7.56, 10.74) |
| **pAMR2 or pAMR3** | 80.0% (60.9%, 91.1%) | 92.7% (92.1%, 93.2%) | 10.96 (8.89, 13.52) |

**Abbreviations:** AMR, antibody mediated rejection; dd-cfDNA, donor-derived cell-free DNA; LR+, positive likelihood ratio

# Supplemental Table 7: dd-cfDNA performance characteristics for antibody mediated rejection at 0.20% and 0.50% thresholds for dd-cfDNA samples collected the same day as biopsy

| **AMR performance characteristics; 0.20% threshold** | | | |
| --- | --- | --- | --- |
| **AMR classification** | **Sensitivity** | **Specificity** | **LR+** |
| **Any AMR** | 48.6% (37.2%, 60.0%) | 87.2% (86.2%, 88.0%) | 3.79 (2.94, 4.87) |
| **pAMR1 (H+), pAMR2, pAMR3** | 55.3% (41.2%, 68.6%) | 87.1% (86.1%, 88.0%) | 4.28 (3.28, 5.58) |
| **pAMR2 or pAMR3** | 60.0% (31.3%, 83.2%) | 86.8% (85.8%, 87.7%) | 4.54 (2.72, 7.57) |
|  | | | |
| **AMR performance characteristics; 0.50% threshold** | | | |
| **AMR classification** | **Sensitivity** | **Specificity** | **LR+** |
| **Any AMR** | 37.1% (26.8%, 48.9%) | 94.3% (93.6%, 94.9%) | 6.46 (4.67, 8.93) |
| **pAMR1 (H+), pAMR2, pAMR3** | 46.8% (33.3%, 60.8%) | 94.2% (93.5%, 94.8%) | 8.07 (5.84, 11.16) |
| **pAMR2 or pAMR3** | 60.0% (31.3%, 83.2%) | 93.9% (93.3%, 94.6%) | 9.90 (5.90, 16.60) |

**Abbreviations:** AMR, antibody mediated rejection; dd-cfDNA, donor-derived cell-free DNA; LR+, positive likelihood ratio

# Supplemental Table 8: Patient level dd-cfDNA performance characteristics for antibody mediated rejection at 0.20% and 0.50% thresholds

| **AMR performance characteristics; 0.20% threshold** | | | |
| --- | --- | --- | --- |
| **AMR classification** | **Sensitivity** | **Specificity** | **LR+** |
| **Any AMR** | 59.5% (50.3%-68.2%) | 81.2% (79.4%-82.9%) | 3.17 (2.65-3.79) |
| **pAMR1 (H+), pAMR2, pAMR3** | 69.1% (57.9%-78.5%) | 81.2% (79.4%-82.8%) | 3.67 (3.07-4.38) |
| **pAMR2 or pAMR3** | 83.3% (64.1%-93.3%) | 80.9% (79.1%-82.5%) | 4.35 (3.56-5.32) |
|  | | | |
| **AMR performance characteristics; 0.50% threshold** | | | |
| **AMR classification** | **Sensitivity** | **Specificity** | **LR+** |
| **Any AMR** | 48.2% (39.2%-57.4%) | 93.3% (92.1%-94.3%) | 7.17 (5.56-9.23) |
| **pAMR1 (H+), pAMR2, pAMR3** | 61.5% (50.1%-71.7%) | 93.3% (92.1%-94.3%) | 9.13 (7.15-11.66) |
| **pAMR2 or pAMR3** | 79.2% (59.5%-90.8%) | 92.9% (91.7%-94.0%) | 11.15 (8.59-14.46) |

Includes endomyocardial biopsy and dd-cfDNA samples collected at any time post-transplant

**Abbreviations:** AMR, antibody mediated rejection; dd-cfDNA, donor-derived cell-free DNA; LR+, positive likelihood ratio

# Supplemental Figure 1: Antibody mediated rejection rates by dd-cfDNA positivity in patients with abnormal graft function

**
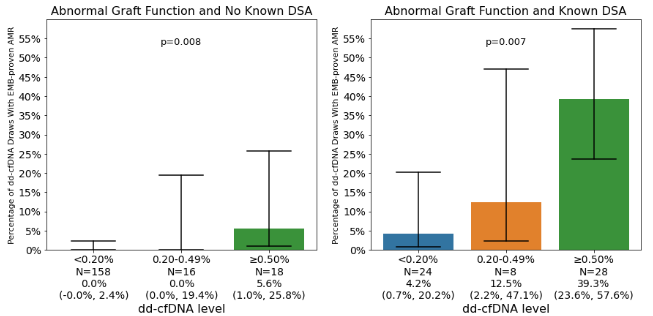
**

Analysis included only endomyocardial biopsies and dd-cfDNA samples collected ≥55 days post-transplant.

**Abbreviations:** AMR, antibody mediated rejection; dd-cfDNA, donor-derived cell-free DNA; DSA, donor specific antibody

# Supplemental Figure 2: Antibody mediated rejection [pAMR1 (H+), pAMR2, or pAMR3] rates by dd-cfDNA positivity in select clinical scenarios

**
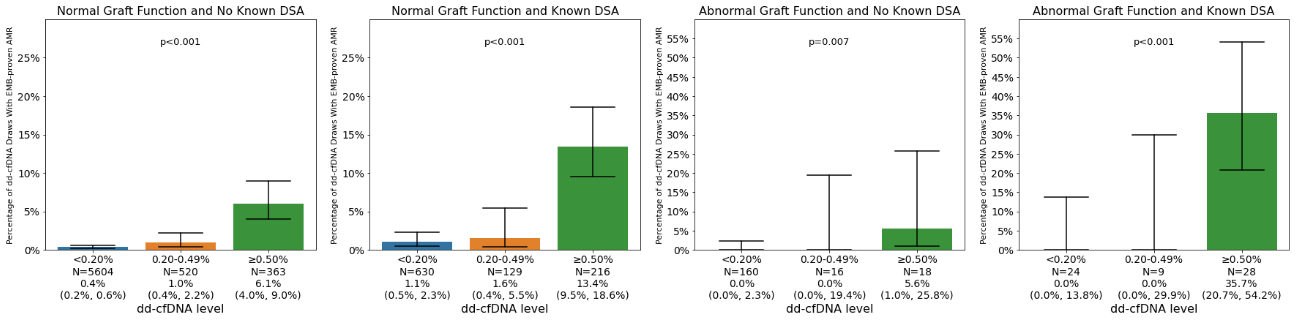
**

Analysis included only EMBs and dd-cfDNA samples collected ≥55 days post-transplant.

**Abbreviations:** AMR, antibody mediated rejection; dd-cfDNA, donor-derived cell-free DNA; DSA, donor specific antibody

# Supplemental Figure 3: Antibody mediated rejection rates by dd-cfDNA positivity including samples collected <55 days post-transplant

**
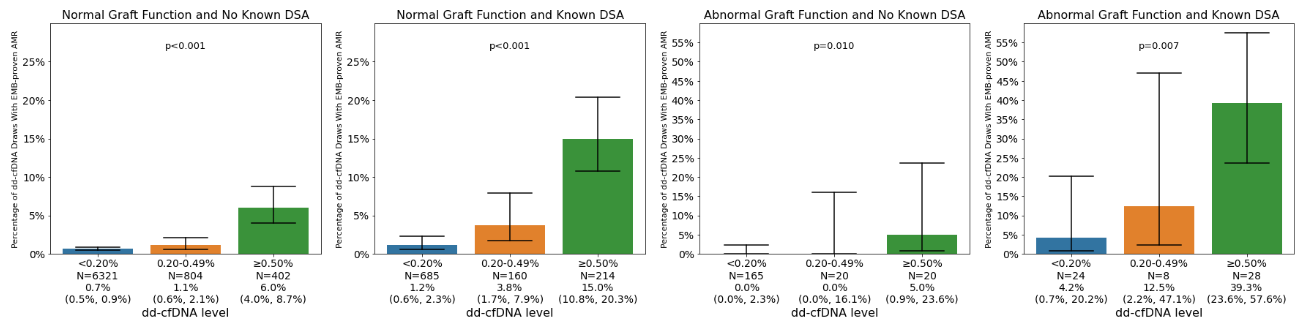
**

**Abbreviations:** AMR, antibody mediated rejection; dd-cfDNA, donor-derived cell-free DNA; DSA, donor specific antibody

# Supplemental Figure 4: Decline in dd-cfDNA following antibody mediated rejection or acute cellular rejection episode


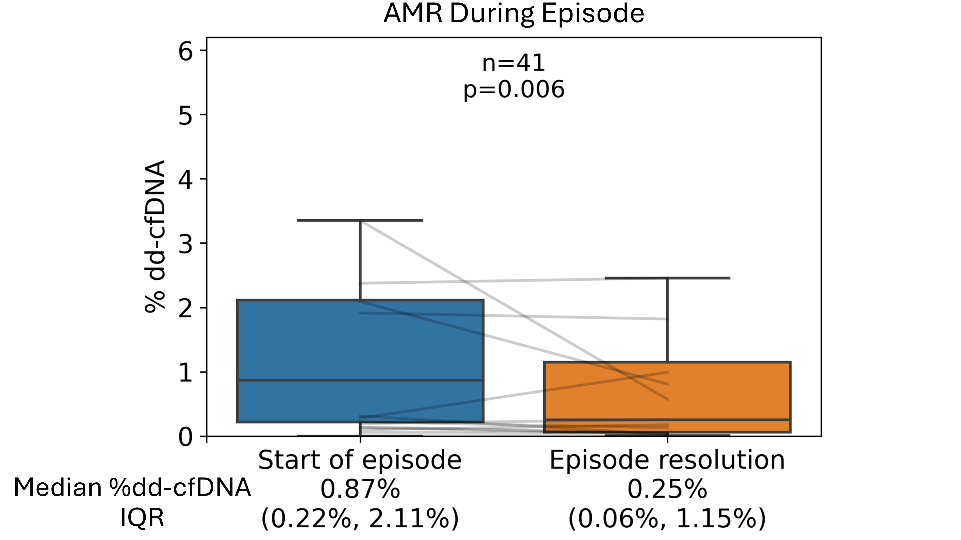
**
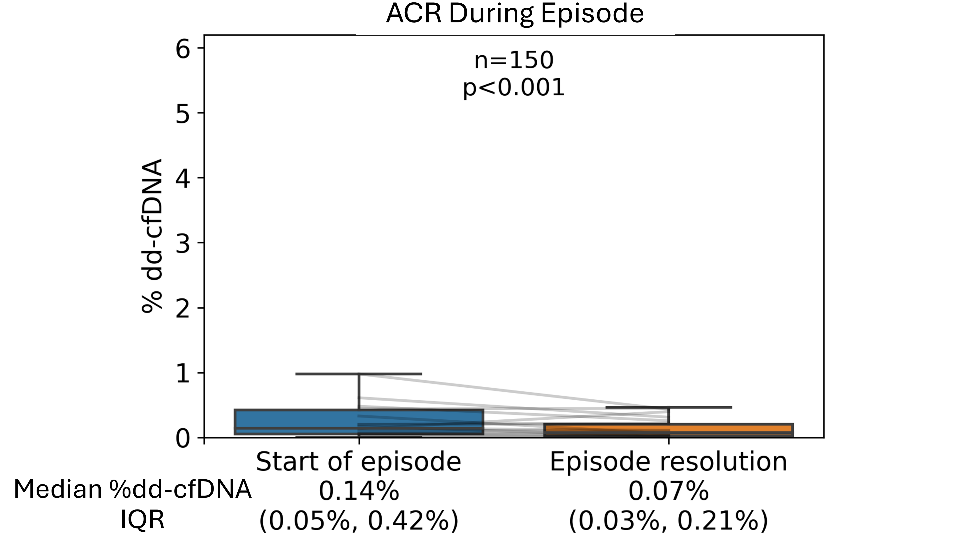
**

Includes all dd-cfDNA paired with start of a rejection episode and only endomyocardial biopsies and dd-cfDNA samples collected ≥55 days post-transplant

**Abbreviations:** ACR, acute cellular rejection; AMR, antibody mediated rejection; dd-cfDNA, donor-derived cell-free DNA
